# Transplantation of iPS cell-derived neural progenitors overexpressing SDF-1α increases regeneration and functional recovery after ischemic stroke

**DOI:** 10.18632/oncotarget.22180

**Published:** 2017-10-31

**Authors:** Monica Chau, Todd C. Deveau, Mingke Song, Zheng Z. Wei, Xiaohuan Gu, Shan Ping Yu, Ling Wei

**Affiliations:** ^1^ Department of Anesthesiology, Emory University School of Medicine, Atlanta, GA, USA

**Keywords:** ischemic stroke, iPS cells, neural progenitor cells, SDF-1, functional recovery

## Abstract

Ischemic stroke is a leading cause of human death and disability while clinical treatments are limited. The adult brain possesses endogenous regenerative activities that may benefit tissue repair after stroke. Trophic factors such as stromal cell-derived factor 1 alpha (SDF-1α) are upregulated in the ischemic brain, which promote endogenous regeneration. The regenerative response, however, is normally insufficient. Transplantation of exogenous cells has been explored as regenerative therapies. One promising cell type for transplantation is induced pluripotent stem (iPS) cells which are cells genetically reprogrammed from adult somatic cells. We hypothesized that transplanting neural progenitor cells derived from iPS cells (iPS-NPCs) could provide cell replacement and trophic support. The trophic factor SDF-1α was overexpressed in iPS-NPCs by lentiviral transduction to test if SDF-1α could increase regeneration in the ischemic brain. These SDF-1α-iPS-NPCs were differentiated *in vitro* to express mature neuronal and synaptic markers. Differentiated cells expressed functional Na^+^ and K^+^ channels, and fired action potentials. In the oxygen glucose deprivation (OGD) test, SDF-1α-iPS-NPCs survived significantly better compared to control iPS-NPCs. In mice subjected to focal cerebral ischemia in the sensorimotor cortex, iPS-NPCs and SDF-1α-iPS-NPCs were intracranially transplanted into the ischemic cortex 7 days after stroke. Neuronal differentiation of transplanted cells was identified using NeuN 14 days after transplantation. Mice that received SDF-1α-iPS-NPCs had greater numbers of NeuN/BrdU and Glut-1/BrdU co-labeled cells in the peri-infarct area and improved locomotion compared to the control iPS-NPC transplantation. Thus, SDF-1α upregulation in transplanted cells may be a therapeutic strategy to enhance endogenous neurovascular repair after ischemic stroke in adult mice.

## INTRODUCTION

Ischemic stroke is a leading cause of death and disability in the U.S and worldwide. Despite its prevalence, there is only one FDA-approved treatment for ischemic stroke, tissue plasminogen activator (tPA). However, its efficacy is restricted to a narrow therapeutic window of 4.5 hrs after stroke so less than 5% of stroke patients have benefited from the thrombolytic treatment [1, 2]. Many candidate neuroprotective drugs to treat stroke in the acute phase have failed to show benefits in clinical trials. Without effective treatments, surviving stroke patients often have neurologic impairments and disabilities for months to years after stroke [3, 4]. Alternative strategies targeting the delayed regenerative phase have drawn increasing attention in hopes of repairing damaged brain tissues and improving functional recovery.

One avenue for regenerative therapy is through stem cell transplantation. Induced pluripotent stem (iPS) cells are derived from somatic cells and have great potential for autologous transplantation which would circumvent host immune rejection [5–8]. Using iPS cells also sidesteps the ethical limitations of obtaining pluripotent cells from human embryos. Importantly, these pluripotent stem cells can be differentiated into any neural cell type including oligodendrocytes, astrocytes, and electrophysiologically functional neurons [9–11]. Along with these advantages, we proposed that an effective stem cell therapy should utilize both exogenous and endogenous regenerative mechanisms for optimal tissue repair and functional recovery. In this investigation, we explore the effect of transplanted neural progenitors derived from iPS cells (iPS-NSCs) on tissue repair and their abilities to enhance endogenous regeneration. Furthermore, we transplanted genetically modified iPS cells to overexpress the chemoattractive factor, stromal-cell-derived factor-1α (SDF-1α) for the purpose of increasing neurogenesis and angiogenesis to the lesion site for additional tissue repair and functional recovery.

In the regenerative niches such as the subgranular zone (SGZ) of the hippocampus and the subventricular zone (SZV), the adult brain has the ability to produce new progenitor cells that can become neurons and non-neuronal cells. After brain injury, this regenerative mechanism is stimulated by increased regenerative genes and trophic factors [12]. However, the endogenous response does not have sufficient regenerative capacity to fully repair damaged ischemic tissue [13]. SDF-1α is a potent chemoattractive factor that is highly upregulated in the infarct after ischemic stroke [14, 15]. Its expression peaks at 7 to 14 days post-ischemia [16]. SDF-1α signaling recruits neural progenitors from the subventricular zone (SVZ) and endothelial progenitor cells to the injured area promoting local angiogenesis and neurogenesis [15, 17]. Moreover, SDF-1 has been shown to be able to stimulate neurite growth of retinal ganglion cells and axon regeneration after optic nerve injury [18]. In the present investigation, we hypothesized that transplanted iPS-NPCs with upregulated SDF-1α would promote an increase of neuronal and vascular progenitors to the ischemic region and improve the therapeutic benefits of the cell-based therapy.

## RESULTS

### SDF-1α expression during neuronal differentiation of iPS cells

**Figure 1:**
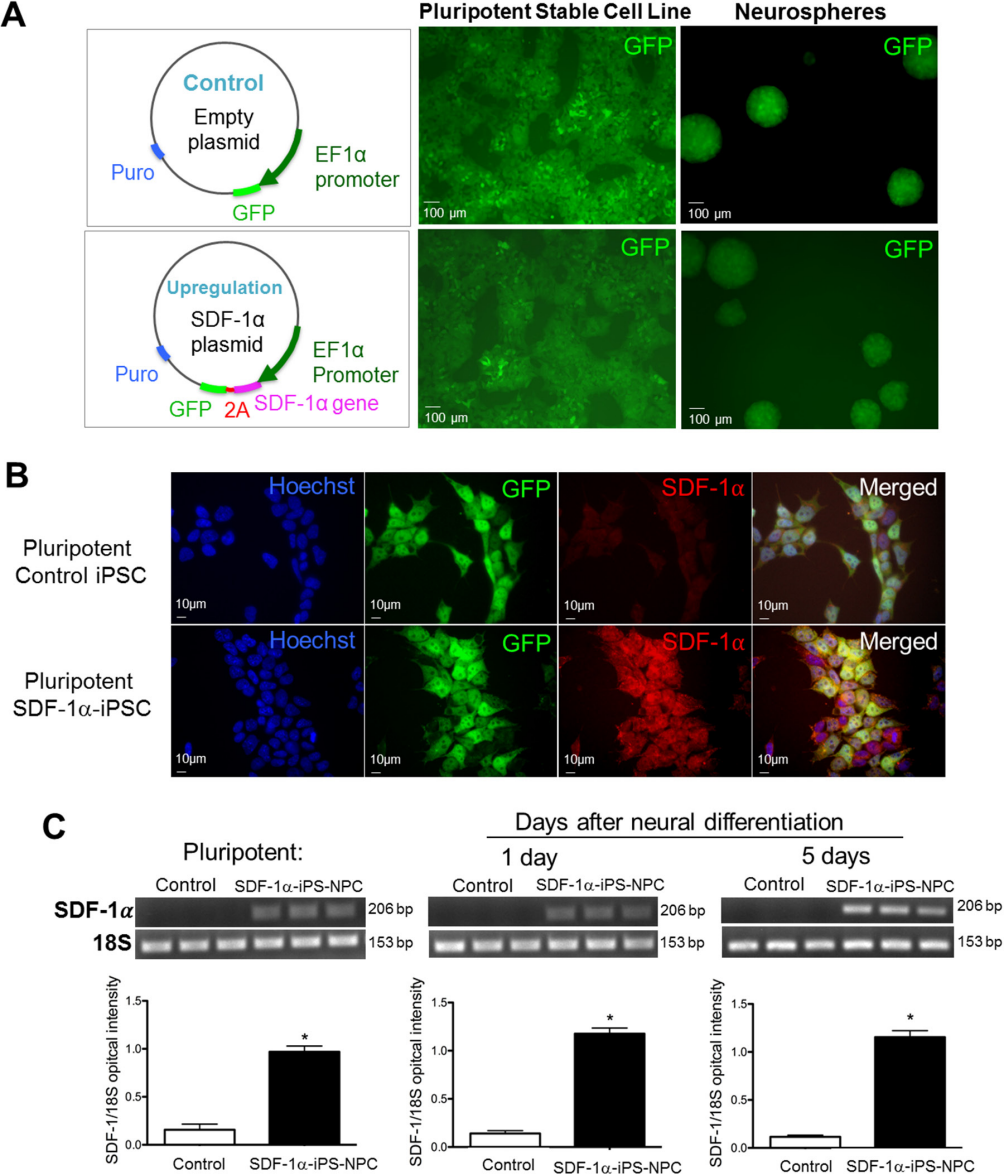
SDF-1α expression before and after neuronal differentiation of iPS cells **(A)** Lentiviral vectors were used for SDF-1α ectopic overexpression. The GFP green fluorescent in naïve and neurospheres shows successful virus transfection. **(B)** Immunocytochemical images of progenitor cells 4 days after neural induction induced by the retinoic acid protocol. In empty vector control cells, SDF-1α staining revealed basal level of endogenous SDF-1α expression. Transfection of SDF-1α containing vectors increased the expression of SDF-1α. **(C)** PCR analysis demonstrated that SDF-1α transfected cells had significantly greater SDF-1α expression compared to controls throughout the pluripotent, progenitor (1d after harvest), and neuronal stages (5d after harvest) of differentiation.

Mouse iPS cells were subjected to lentivirus transduction containing the control plasmid or the SDF-1α gene overexpression plasmid (Figure 1A). The cells were selected via puromycin resistance to create a stable cell line. As a result, both the control and SDF-1α stable cell populations expressed GFP and retained GFP expression during neuronal differentiation in neurospheres (Figure 1A). During the pluripotent stage (Figure 1B) and after neural differentiation with the retinoic acid (RA) protocol [10], both SDF-1α-upregulated and control lines retained GFP expression allowing for ease of cell visualization and quantification in tissues after transplantation. PCR analysis demonstrated that SDF-1α cells (SDF-1ɑ-iPS-NPCs) had significantly greater SDF-1α expression compared to controls (Figure 1C). The ectopic upregulation of SDF-1α was maintained throughout the pluripotent, progenitor (1 day after harvest), and neuronal stages (5 days after harvest) of the neuronal differentiation (Figure 1C). Taken as a whole, SDF-1 expression is higher in the cultures that were infected with virus. Our results do not differentiate between neurons and other cell types in the culture expressing upregulated SDF-1.

### SDF-1ɑ-iPS-NPCs differentiated into functional neurons *in vitro*

**Figure 2:**
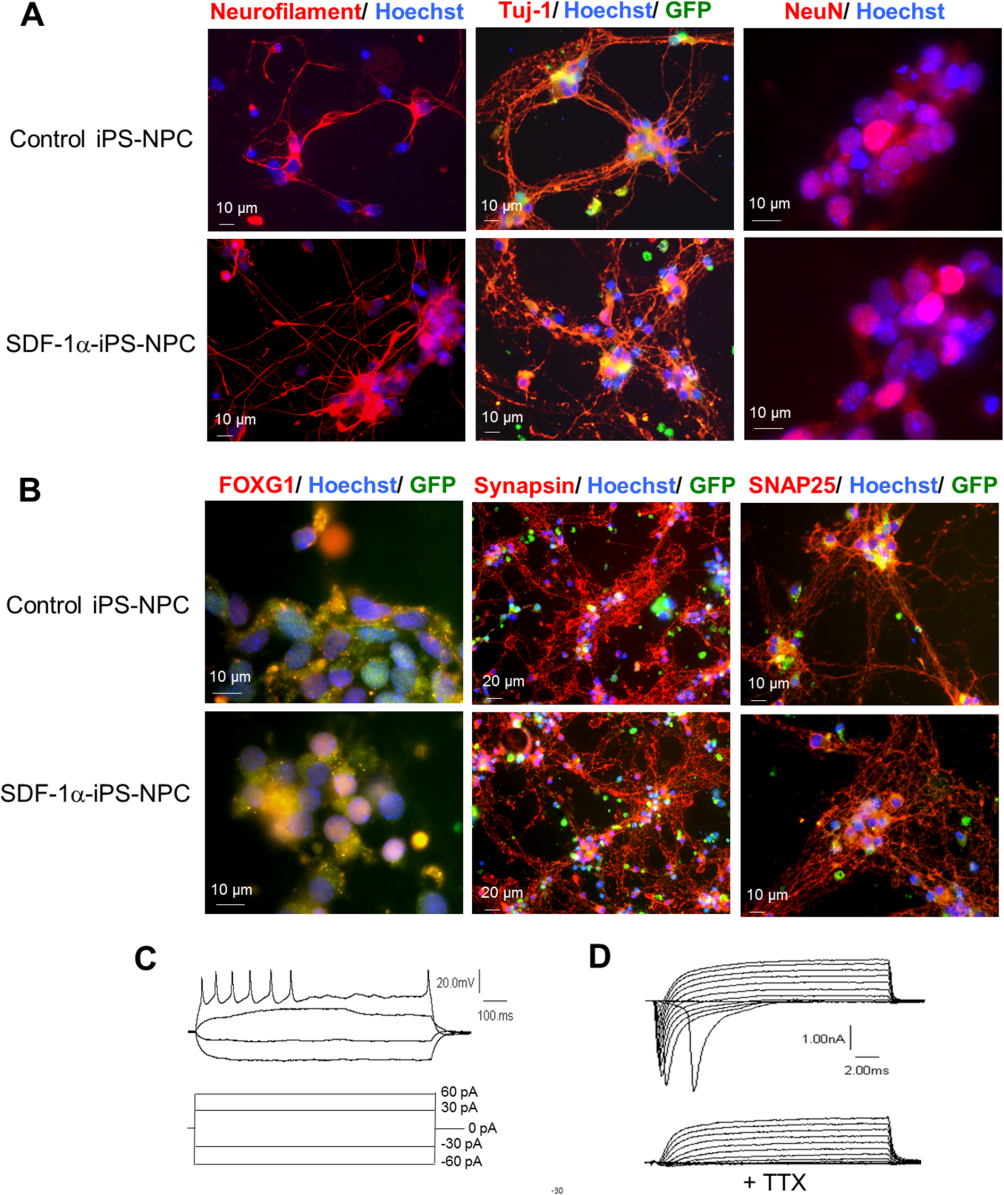
SDF-1ɑ-iPS-NPCs differentiated into functional neurons *in vitro* **(A)** After neuronal differentiation, SDF-1α cells and control cells both expressed the mature neuronal markers, Tuj-1, neurofilament, and NeuN 5 days after differentiation. **(B)** Both cell lines also exhibited the forebrain marker, FoxG1 and the synaptic markers, synapsin and SNAP25**. (C** and **D)** SDF-1α iPS-derived neurons exhibited evoked action potentials (C), inward sodium currents and outward potassium currents (D). Application of tetrodotoxin (TTX) attenuated the inward sodium current (D).

Before differentiation, both the control and SDF-1ɑ iPS cells expressed the pluripotent marker Oct3/4 (data not shown). After the RA neural induction protocol, the cells were harvested and plated for terminal differentiation on PDL/laminin-coated dishes and continuously cultured for 5 days. After the 5 days of terminal differentiation, immunocytochemical staining detected the neuronal markers, neurofilament, Tuj-1, as well as NeuN in SDF-1ɑ-iPS-NPCs and control iPS-NPCs (Figure 2A). Both cell lines exhibited the forebrain marker, FoxG1 and the synaptic markers, synapsin and SNAP25 (Figure 2B). Further, fast inward Na^+^ currents, outward K^+^ currents, and evoked action potentials were recorded in these cells using whole cell recordings (Figure 2C). Tetrodotoxin (TTX) selectively blocked the voltage-gated inward Na^+^ current (Figure 2D).

### Expression of SDF-1α increased cell survival after a hypoxia insult *in vitro*

**Figure 3:**
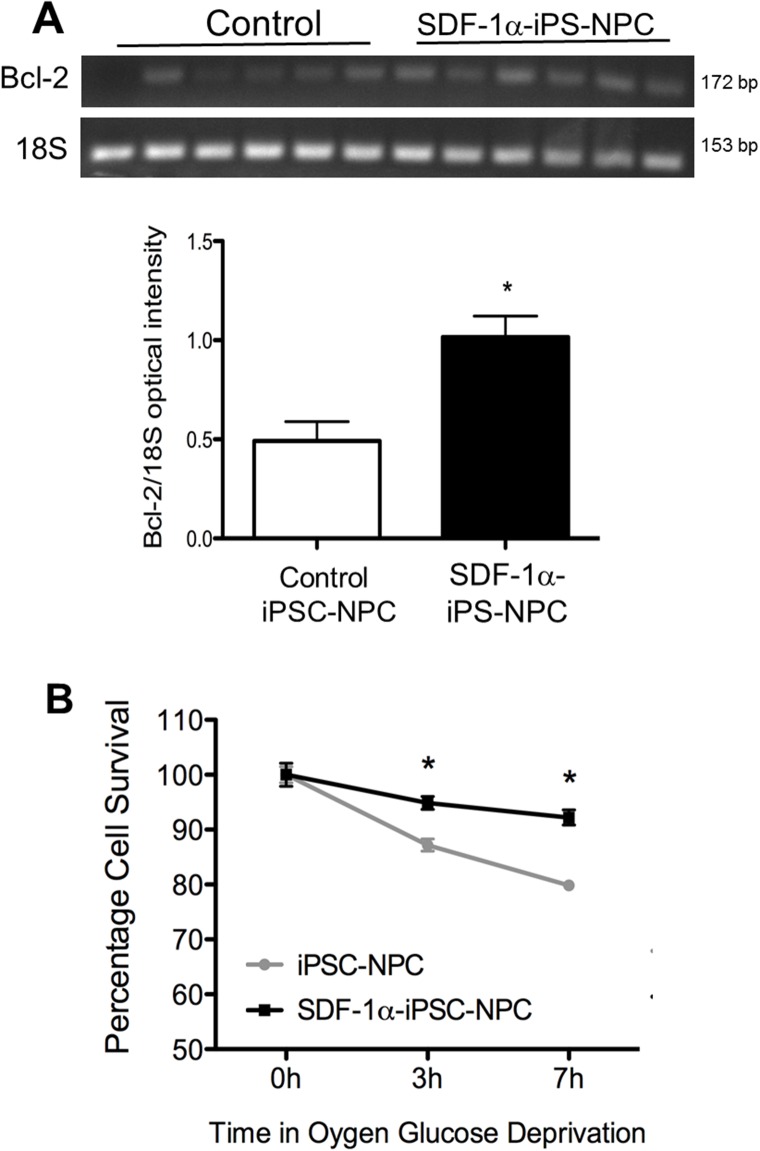
SDF-1α expression increased cell survival after *in vitro* ischemic insult **(A)** PCR analysis showed that Bcl-2 was upregulated in SDF-1α cells compared to control cells (n=6, ^*^. p=0.0045). **(B)** To test survival, cells were challenged with oxygen-glucose deprivation (OGD) in a hypoxia chamber for 3 or 7 hrs followed by 12h of “reperfusion” in normoxia. Cell viability was then measured using MTT assay. SDF-1ɑ-iPS-NPCs exhibited greater viability after OGD compared to control cells (n=4-6, ^*^. p=0.0006). The mean and standard error of the mean are plotted in the line graph.

Since SDF-1ɑ plays a role in cell survival, we tested a pro-survival marker, Bcl-2. PCR analysis showed that Bcl-2 expression was upregulated in SDF-1ɑ-iPS-NPCs compared to iPS-NPC control cells (Figure 3A). We thus examined whether or not SDF-1ɑ played a role in cell survival under ischemia-like conditions. iPS-derived neurons after terminal differentiation were exposed to oxygen glucose deprivation (OGD), an *in vitro* model of ischemia. The OGD insult was carried out in a hypoxia chamber with 0.1% O_2_ for 3 or 7 hrs followed by 12h of reoxygenation in normoxia. Viability in the OGD experiments was determined using the MTT assay. Compared to control iPS-NPCs, SDF-1ɑ-iPS-NPCs exhibited greater viability after OGD (Figure 3B).

### SDF-1α expression and neuronal differentiation of SDF-1ɑ-iPS-NPCs *in vitro* and in the post-ischemic brain

**Figure 4:**
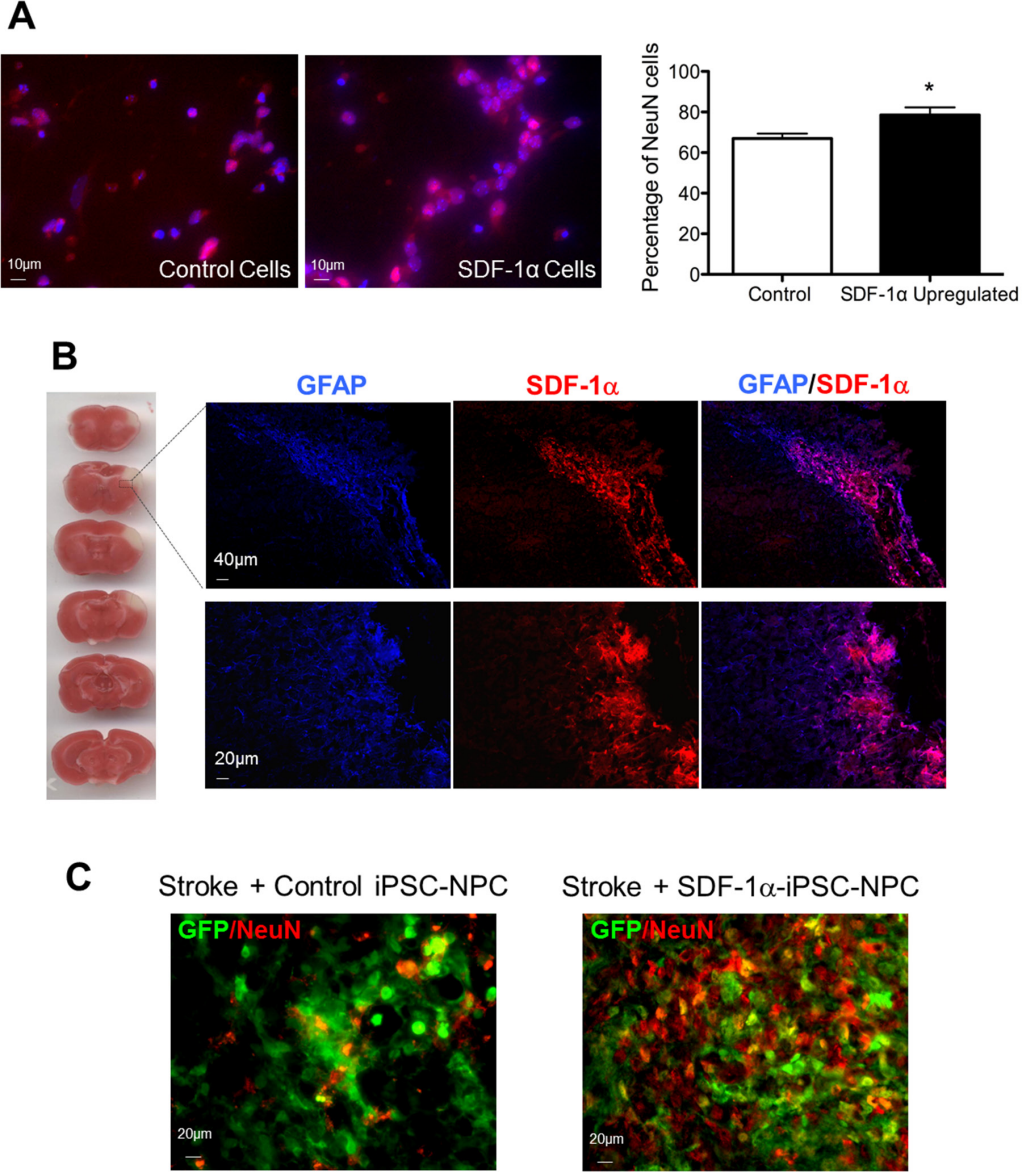
Expression of SDF-1α and neuronal differentiation of SDF-1ɑ-iPS-NPCs in the post-ischemic brain **(A)** In an *in vitro* assay, neurally induced SDF-1α-iPS-NPCs showed an increase in differentiation into NeuN-positive cells compared to control iPS-NPCs (n=6, ^*^. p=0.037). The mean and standard error of the mean are plotted. **(B)** TTC staining (red) shows the cortical damage (white) in the sensorimotor cortex of the focal ischemic stroke model 24 hrs after the insult. Seven days after stroke, SDF-1α expression in the cortex was detected using immunohistochemical staining in different mice in the peri-infarct area (rectangular frame). These mice did not receive transplants. Here, TTC staining and immunofluorescence were in different mouse tissues. Many SDF-1 positive cells were also GFAP positive, consistent with astrocyte accumulation in the region at this time. **(C)** Two weeks after transplantation, transplantediPS-NPCs or SDF-1α-iPS-NPCs showed NeuN expression visualized with GFP/NeuN co-labeling in the peri-infarct area.

We tested if the ectopic overexpression of SDF-1α conferred advantages to the cells besides increased cell survival. After applying the neuronal differentiation protocol *in vitro*, SDF-1α-iPS-NPCs showed more differentiation into NeuN-positive cells compared to control iPS-NPCs (Figure 4A). There were significantly more NeuN-positive cells derived from SDF-1 overexpressing cells compared to control cells (66.9% versus 78.5% of NeuN-positive cells from control and SDF-1 cells, respectively, p<0.05) (Figure 4A).

In our focal ischemia model, stroke was targeted to the right sensorimotor cortex of the mouse [9, 19]. The endogenous SDF-1α expression was detected in the infarct area 7 days after stroke (Figure 4B). SDF-1α has been shown to be upregulated in neurons, vessels, and astrocytes after ischemia [20, 21]. In our experiment, many SDF-1α positive cells were co-labeled with GFAP staining after focal ischemia (Figure 4B).

GFP-labeled iPS-NPCs and SDF-1α-iPS-NPCs (100,000 or 300,000 cells as low and high dose groups) were intracranially grafted into the peri-infarct region 7 days after stroke in the regenerative phase of stroke [20, 21]. This transplantation time point was selected to avoid the acute excitotoxic/inflammatory factors and brain edema during early days after stroke and targeted to improve chronic regeneration and tissue repair. Two weeks after transplantation, transplanted GFP-labelediPS-NPCs and SDF-1α-iPS-NPCs showed differentiation into GFP/NeuN double-positive cells visualized in the peri-infarct area (Figure 4C).

### Transplantation of SDF-1α-iPS-NPCs increased regenerative activities in the post-stroke brain

**Figure 5:**
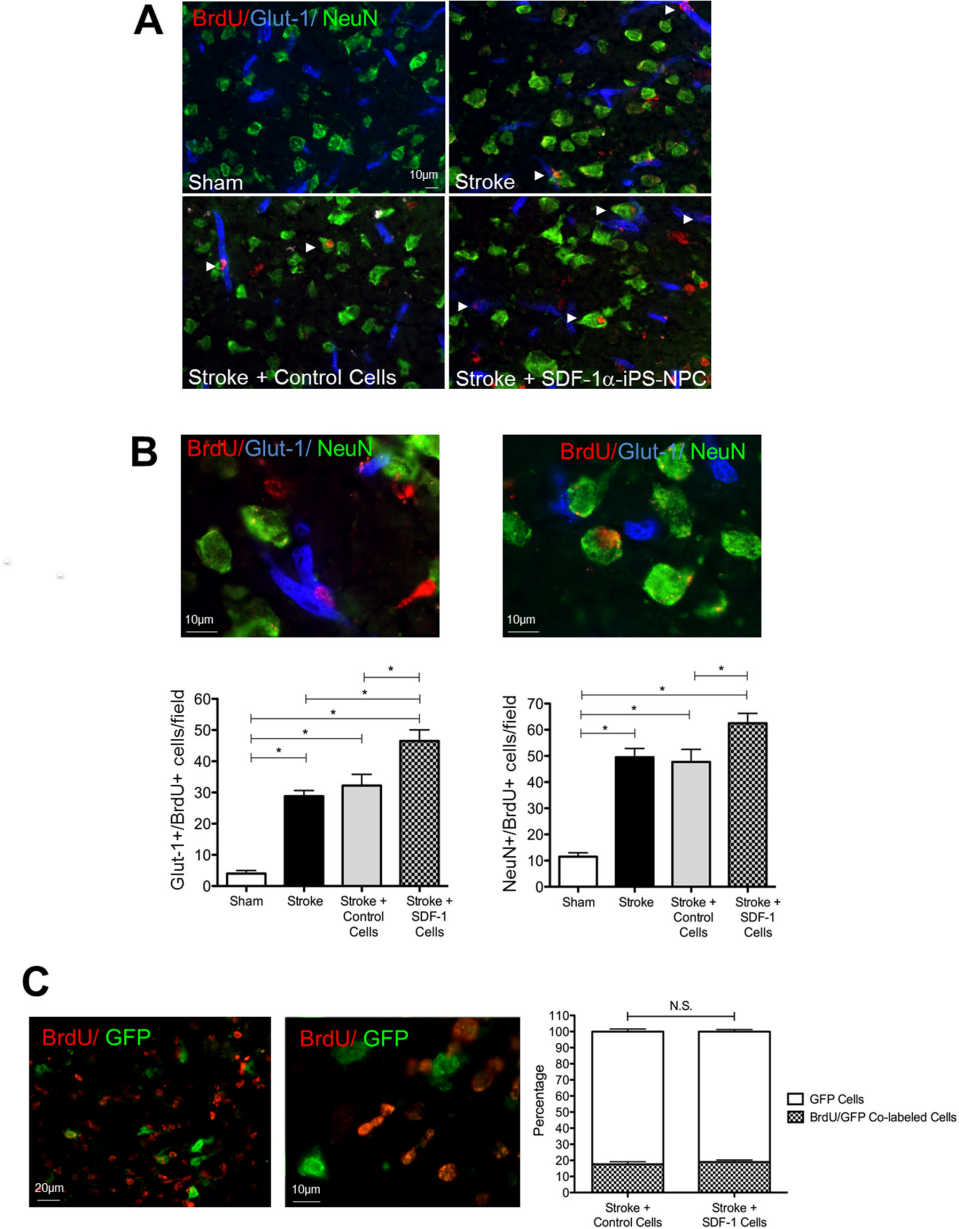
Transplantation of SDF-1α-iPS-NPCs increased regenerative activities in the post stroke brain **(A)** In immunohistochemical staining, co-labeled NeuN/BrdU cells and Glut-1/BrdU cells (arrows) were used to detect newly formed neurons and endothelial cells in the peri-infarct area. **(B)** An enlarged image demonstrates the colocalization of NeuN (pink) and BrdU (white). **(C)** Pictures were captured from 4 areas 700-900 μm from the edge of the injury. Six tissue sections from each animal brain were quantified. The graphs here reflect the average number of co-labeled NeuN/BrdU and Glut-1/BrdU cells in the optical field. There were significantly more NeuN/BrdU-positive cells (n=10; ^*^. p=0.015) and Glut-1/BrdU-positive (n=10; ^*^. p=0.0008) in the SDF-1α cell transplantation group compared to the sham, stroke only, and stroke + control cells groups, C. As a control, the majority of GFP-positive transplanted cells did not co-label with BrdU and there was no difference in proliferation between iPS-NPCs and SDF-1α-iPS-NPCs (n=4 per group, ^*^. p=0.5) after transplanting these cells *in vivo*, suggesting that the increased regeneration observed was mainly due to endogenous cells. Mean ± SEM.

To label newly formed cells, the mice were injected with BrdU (50 mg/kg/day i.p) on the day of transplantation until the day of euthanasia/tissue collection. Coronal brain sections were analyzed for neurogenesis and angiogenesis in the peri-infarct area 14 days after cell transplantation. We quantified the number of co-labeled NeuN/BrdU cells and Glut-1/BrdU cells for newly formed neurons and endothelial cells respectively in the peri-infarct area of the brain (Figure 5A). Pictures were captured from 4 areas approximately 700-900 μm from the edge of the injury. Six tissue sections from each animal brain were quantified. The graphs here reflect the total number of co-labeled NeuN/BrdU and Glut-1/BrdU cells from each animal. There were significantly more Glut-1/BrdU-positive and NeuN/BrdU-positive cells in the stroke plus SDF-1α-iPS-NPCs transplantation group compared to the sham, stroke only, and stroke plus control iPS-NPCs groups, (Figure 5A-5C). As a control, the majority of GFP-labeled transplanted cells did not co-label with BrdU (∼80% for both iPS-NPCs and SDF-1α-iPS-NPCs, no significant difference).

### Transplantation of iPS-NPCs increases functional recovery

**Figure 6:**
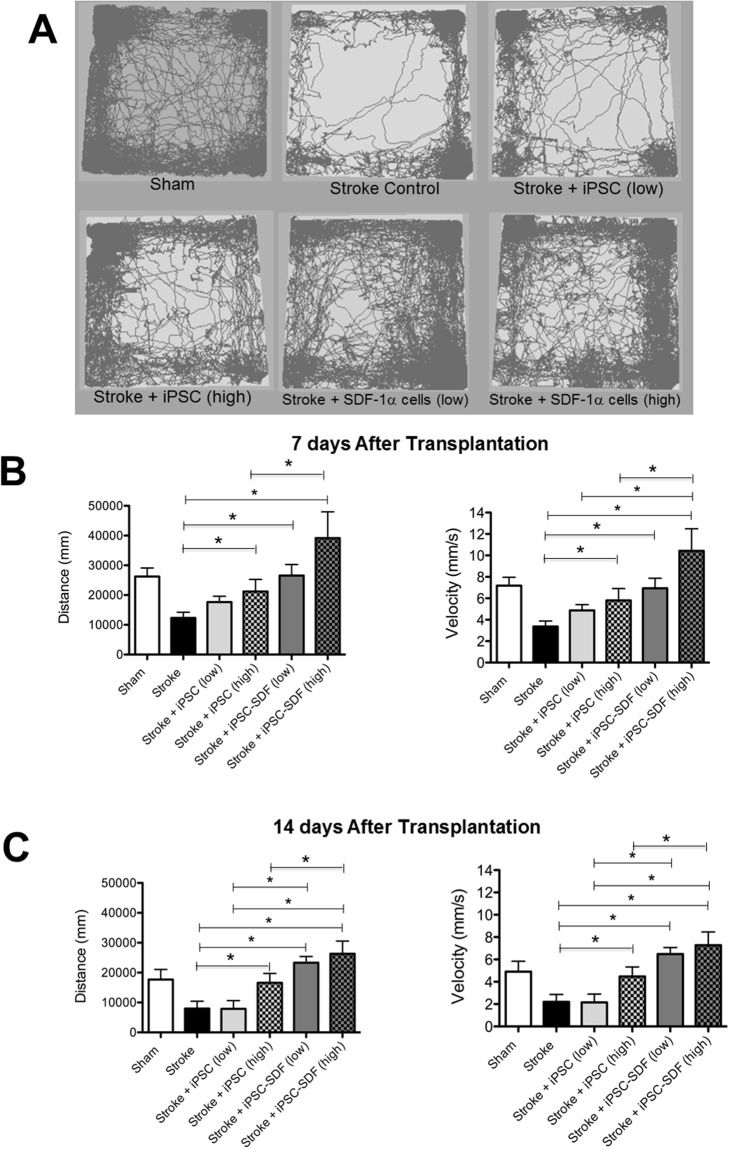
Transplantation of iPS-NPCs increased locomotor functional recovery after stroke **(A)** Using a behavior tracking system, the distance and the velocity of walking during a one- hour period was recorded and analyzed (one-way ANOVA with Bonferroni’s post-hoc correction for multiple comparisons). **(B)** At 7d after transplantation, mice with stroke + high dose of iPS-NPC or SDF-1ɑ-iPS-NPC transplantation exhibited significantly more movement compared to stroke only animals (n=5, ^*^. p<0.05). There was also significant difference in the velocity of movement between stroke and stroke + high dose iPS-NPC or SDF-1ɑ-iPS-NPC group (p<0.05). iPS-NPCs with SDF-1ɑ overexpression showed even more increased locomotion compared to regular cells (^*^. p<0.05). **(C)** At 14d after transplantation, there were significant differences between stroke and stroke plus cell transplantation (^*^. p<0.05), except the lose dose iPS-NPC group (n=5). Similar changes were seen with walking velocity (n=5, ^*^. p<0.05).

The goal of regenerative stroke therapies is to improve functional recovery. We compared low dose (100,000 cells) and high dose (300,000 cells) transplantations of iPS-NPCs and SDF-1ɑ-iPS-NPCs into the ischemic brain. Using a behavior tracking system, the walking distance and the velocity of movement during a one-hour period were analyzed (Figure 6A). At 7 and 14 days after cell transplantation, the stroke mice that received high dose iPS-NPCs showed significant improvements in locomotion (Figure 6B). Furthermore, the transplantation with SDF-1ɑ-iPS-NPCs exhibited even more enhanced movement activities (Figure 6B). The low dose transplantation group did not show significant improvements in locomotion at 7 days, but significant benefits of low and high dose transplantations into mice were seen at 14 days after stroke (Figure 6C). SDF-1ɑ-iPS-NPC transplantation again showed enhanced recovery compared to control cells.

## DISCUSSION

This investigation tested the hypothesis that overexpressing SDF-1α in transplanted iPS cell-derived NPCs would promote endogenous regenerative mechanisms by increasing more neural and vascular progenitor cells at the ischemic injury site. This strategy combines exogenous and endogenous regenerative mechanisms to optimize the therapeutic benefits. We showed that iPS cells with SDF-1α overexpression retained the ability to differentiate into electrophysiologically functional neurons *in vitro*. One of the benefits of SDF-1α expression in the iPS-NPCs is an increased tolerance to the OGD insult. Furthermore, after transplantation into the ischemic brain, GFP-labeled control and SDF-1α-iPS-NPCs can differentiate into neurons in the peri-infarct area. To support our hypothesis, transplantation of SDF-1α-iPS-NPCs increased the number of endogenous cells at the peri-infarct cortex, showing increased neurogenesis and angiogenesis, and improved behavioral recovery. Our data demonstrated dual benefits of transplanting SDF-1α-iPS-NPCs into the injury including contributing exogenous cells that could differentiate into neurons and also enhancing endogenous regeneration after ischemic stroke.

SDF-1α plays pleiotropic roles in regeneration after stroke particularly in progenitor cell recruitment and cell survival [15, 22]. The ectopic upregulation of SDF-1α has been previously used as a tool to study chemotaxis in stroke, myocardial infarction, and inflammation [23–25]. SDF-1α expression is overexpressed endogenously in the ischemic region after stroke as a mechanism of self-repair [15, 21]. Upon ischemia, the HIF-1α oxygen-sensing pathway is activated which increases SDF-1α expression [26]. The result is a gradient expression of SDF-1α in the infarct and peri-infarct areas with SDF-1α expression strongest in the core [14, 15]. Consistent with the literature, we observed an astrocytic SDF-1α expression 7 days after stroke in our focal ischemic stroke model [16, 27, 28]. We reported that transplanted cells such as bone marrow mesenchymal stem cells (BMSCs) showed an effect of increasing the expression of SDF-1a in the ischemic brain [29]. In addition to the endogenous expression of SDF-1α, our goal was to provide the peri-infarct area with supplemental SDF-1α since the endogenous regenerative mechanisms do not sufficiently repair tissue after stroke.

To support our hypothesis that iPS-NPCs have the potential to provide neuronal cell replacement, we differentiated the pluripotent cells down the neural lineage. The differentiated control and SDF-1α iPS cells displayed neuronal phenotypes such as the expression of mature neuronal markers, NeuN, Tuj-1, and neurofilament demonstrating that neuronal differentiation is not impeded by SDF-1α upregulation in the iPS cells. Furthermore, control and SDF-1α iPS cells can be differentiated into functional neurons exhibiting action potentials and functional K^+^ and Na^+^ channels. We showed before that mouse iPS cells subjected to the 4-/4+ differentiation protocol can express NeuN and generate action potential in about 7 days after the induction [30]. The differentiated cells also expressed FOXG1, a forebrain-specific marker appropriate for replacing cells in the cerebral cortex [31]. Both cell lines expressed synaptic markers, SNAP25 and synapsin after neuronal differentiation suggesting that these cells have the machinery to build synaptic connections.

Another advantage of upregulating SDF-1α was to bolster cell survival [32, 33]. Due to the cytotoxic nature of the stroke injury, the transplantation of exogenous cells can be challenging. Generally only a small percentage of cells survive after transplantation [34, 35]. To test cell survival, the cells were treated with ischemia-like conditions. With the OGD insult, SDF-1α cells showed greater rates of survival. It is probable that SDF-1α was secreted from the cells and bound to the CXCR4 receptor on neighboring cells to inactivate posttranslational cell death mechanisms such as inhibiting BAD, and also promote pro-survival genes such as Bcl-2 [36]. Cells exposed to a hydrogen peroxide (H_2_O_2_) insult exhibited fewer caspase-3-positive cells and more Bcl-2 expression when SDF-1α was added *in vitro* [37]. Furthermore, when SDF-1α protein was injected into the ischemic brain, infarct size decreased and motor function increased suggesting that SDF-1α contributes to neuroprotection and repair in the brain [37].

The cell transplantation was performed in the delayed phase of stroke to supplement the rising SDF-1α expression between 7 and 14 days when regenerative activities occur. This timing also avoided the cytotoxic milieu of the acute phase of stroke. We observed a typical increase in angiogenesis and neurogenesis after stroke [17, 20], but there was an even greater increase of angiogenesis and neurogenesis with SDF-1α-iPS-NPC transplantation. These findings support the role of SDF-1α in regeneration after ischemia [15, 17]. One source of neural progenitors is the SVZ from which the progenitors migrate along the corpus callosum to the cortical infarct in our model [38–40]. Endothelial progenitor cells originate from a different source; they are hematopoietic and disseminate in the body via the circulatory system [41]. Both neural progenitors and endothelial progenitor cells express the SDF-1α receptor, CXCR4 [33, 42]. SDF-1α binds to this receptor on the cell to elicit G-protein coupled receptor cascades activating intracellular pathways for motility and cytoskeletal changes in the cell, such as the phosphorylation of ribosomal S6 kinase (p90^RSK^) involved in neurite outgrowth [43, 44] or the phosphorylation of c-Jun which is involved in cellular migration [45]. The regeneration we observed support the use of SDF-1α-upregulated iPS-NPCs for neurovascular repair [17].

The role of SDF-1 peptides has dichotomous effects in that it can attract regenerative progenitors as well immune cells such as lymphocytes and monocytes as reflecting the functional complexity of the SDF-1 family [46]. This may be modulated by isoform-specific SDF-1 expression. The SDF-1 peptide has several splice variants including SDF-1α and SDF-1β. Evidence suggests that SDF-1β plays a greater role as a chemoattractant for peripheral blood cells such as ciruculating leukocytes into the ischemic area whereas SDF-1α has more of an effect on neuronal plasticity after stroke [16, 47]. This is an example of how one gene can modulate several events after an injury, and further studies are needed to test the inflammatory effect after the transplantation of SDF-1α cells.

Another caveat to introducing SDF-1α iPS-NPCs into the brain is that SDF-1α has also been implicated in tumorigenesis [48, 49]. In our lab, a group of 5 transplanted animals were followed for 4 months after transplantation to see whether or not tumors formed. We analyzed these tissues under H & E staining and no tumor formation was detected at 4 months. Tumorigenesis has been a major concern in pluripotent stem cell transplantation [50]. In this study, we transplanted the cells as differentiated neural progenitors since transplantation of undifferentiated stem cells could lead to tumorigenesis [51, 52] and lineage-restricted stem cells are better candidates [53]. One translational aspect to consider is the cell dosing and the amount of exogenous SDF-1α introduced into the brain. Studies would be needed to optimize the SDF-1α concentration secreted by the cells, possibly using an inducible vector. Also, the cells transplanted would have to be cell-sorted for neural progenitors specifically eliminating any pluripotent cells. Our observations reveal that a small portion of transplanted cells were co-labeled with BrdU indicating that the cells could still be proliferative as neural progenitors or pluripotent cells. We started injecting BrdU on the same day that we transplanted iPS cells (7 days after stroke) to see the effect of the transplant on proliferation activities either on endogenous or transplanted cells. In immunostaining examinations, these BrdU positive cells did not exhibit fragmented nuclear, suggesting they were not damaged cells or cells with DNA fragmentation. Further evaluations of staining for Ki-67, PCNA, CD133, and p53 are needed [54–56] to test for tumorigenic markers.

In this investigation, we also tested the functional recovery of the mice after the transplantation of iPS-NPCs. We demonstrated that mice with iPS-NPCs transplantation and SDF-1α iPS-NPC transplantation had moved significantly greater distances with greater velocity compared to stroke only controls at 14d after transplantation. Based on the angiogenesis and neurogenesis observed with SDF-1α iPS-NPC transplanted mice, the improved functional recovery could be due to neurovascular coupling in the peri-infarct area [17]. Angiogenesis represents a histological correlate to the improved functional recovery by providing new blood flow as support to the injured cells in the peri-infarct area. This improvement on locomotion and overall migration may also be explained by SDF-1α playing a role in modulating the firing patterns of neurons [57–59]. Currently, there has been little research on the effect of SDF-1α on neuronal function, however there are some reports demonstrating SDF-1α modulates the release arginine vasopressin (AVP) in the magnocellular neurons of the supraoptic nucleus (SON) and the paraventricular hypothalamic nucleus. The changes in AVP release could initiate a systemic effect affecting overall movement and health. Another report demonstrates that SDF-1α enhances GABA and glutamate synaptic activity at serotonergic neurons modulating serotonin release [59]. This may be a systemic neurotransmitter correlate to explain improved locomotion.

Collectively, this data is the first to demonstrate that SDF-1α iPS-NPC transplantation enhances the endogenous neurovascular regeneration and locomotor functional recovery after ischemic stroke. Furthermore, iPS-NPC transplantation also has the advantage of providing more neuronal-lineage cells into the injury area. We were able to encourage more regeneration to this area after ischemic stroke and see behavioral improvements supporting the use of trophic factor-enhanced iPS-NPCs for ischemic stroke.

## MATERIALS AND METHODS

### Cell culture

Pluripotent mouse iPS cells were purchased from Stemgent (Cambridge, MA). They were maintained as pluripotent cells in a growth culture medium consisting of 15% ES cell fetal bovine serum (ES Cell FBS, Gibco Life Technologies, Grand Island, NY) in Dulbecco’s Modified Eagle Medium (DMEM; Corning Cellgro, Manassas, VA) with 1% non-essential amino acids, 1% penicillin-streptomycin, 0.1% beta-mercaptoethanol, and 1:10,000 leukemia inhibitory factor (LIF). Cells were passaged with 0.25% trypsin-EDTA when growth was approximately 80% confluent. Cell media was changed if the cells were not confluent enough to passage. Pluripotent cell cultures were grown on mouse embryonic fibroblast (MEF) feeder layers (Millipore, Billerica, MA) immediately after thawing. After approximately 6 passages on MEFs, the cultures were maintained in T-25, or 25cm^2^ flasks pre-coated with 0.15% gelatin. In suspension culture, iPS cells were differentiated with the 4-/4+ (4 days without/4 days with) all trans-retinoic acid (RA, Sigma, St. Louis, MO) protocol in media without LIF [10, 60]. To initiate differentiation, the pluripotent cells were dissociated from the flask with 0.25% trypsin without EDTA. Approximately four million dissociated iPS cells were plated in uncoated/untreated petri dishes for suspension culture in growth media. The cells formed aggregates, or embryoid bodies in suspension and the media was changed every other day throughout the differentiation protocol. Retinoic acid (5 x 10^-7^ M) was added to the media for the last four days of the differentiation protocol [10]. To harvest, the aggregates were dissociated with trypsin-EDTA and run through a cheesecloth filter to dissociate the remaining clumps. 100,000 or 300,000 cells were suspended in 4 μl of SATO and transplanted via 2 x 2 μl injections at 2 sites in the peri-infarct cortex. The injection sites were approximately 3mm lateral to bregma. Cells that were not transplanted were plated in SATO media [61] on PDL/laminin-coated dishes and were allowed to terminally differentiate *in vitro*.

### SDF-1α upregulation plasmids

The SDF-1α upregulation plasmid was created by Dr. Oskar Laur at the Emory University Custom Cloning Core Facility. The human SDF-1α gene was obtained from Addgene (plasmid ^#^12270) and sub-cloned under the human elongation factor 1α (EF1α) promoter in the plasmid, pEGIP (Addgene, plasmid ^#^26777). The SDF-1α upregulation plasmids were created with the backbone, pEGIP which has GFP reporter gene and puromycin resistance for selection. The SDF-1α plasmid was created with the 2A expression system. Control plasmids were empty vectors without the SDF-1α gene and GFP expressed under the IRES system inherent to the pEGIP backbone. The empty plasmid (control) and the SDF-1α plasmid were packaged into lentiviruses.

### Lentivirus production

The plasmids were packaged into lentiviruses by Dr. Xinping Huang at the Emory University Viral Core. HEK 293FT cells (Invitrogen, Waltham, MA) were maintained in growth media (4.5 g/L glucose and L-glutamine containing DMEM supplemented with 10% FBS and 1% Penicillin-Streptomycin) and incubated at 37°C, 5% CO_2_. The HEK 293FT cells were approximately 80% confluent at the time of transfection. The following mixture was prepared: 250 μg of FDS1 or control plasmid + 187.5 μg of pCMVdelta 8.9 + 75 μg of pV-SVG + 12 ml of ddH_2_O + 12.5 ml of 0.5 M Ca_2_Cl + 25 ml of 2x HeBS to total volume 50ml. This solution was shaken and incubated for 20 min at room temperature. Five ml of the mixture was added drop-wise to each dish and the dishes were returned to the incubator. Seven hours post-transfection, the media was replaced with 20 ml of fresh media and incubated for an additional 48 hrs before harvesting. The supernatant containing the lentivirus was collected 2 days after the 48 and 72 hrs post-transfection. The supernatant was centrifuged at 500xg for 5 min at 4°C then passaged through a 0.45 μm low protein binding filter. The supernatant (400 ml total) was loaded into six 70 ml ultracentrifuge tubes and centrifuged at 28,000 rpm for 2 hrs at 4°C in a 45Ti rotor (Beckman, Brea, CA). The virus pellets were resuspended in 500 μl of PBS and incubated on ice for 30 min. The six tubes of resuspended virus were combined and then loaded to a 12 ml SW 41 tube. Three ml of 20% sucrose was added as a cushion then centrifuged at 28,000 rpm for 2 hrs at 4°C in a SW 41 rotor (Beckman). The virus pellet was resuspended in 100 μl of PBS and incubated for 2 hrs at 4°C. The viral titers were 1x10^8^ IU/ml for the control and 1x10^7^ IU/ml for the SDF-1α upregulation plasmid.

### Stable cell line creation

The pluripotent iPS cells were infected with the control or SDF-1α lentivirus to create stable cell lines. Virus was applied to the iPS cells, and incubated for 24 hrs. Twenty-four hours after the application, GFP expression was visually confirmed in control virus and SDF-1α virus cells. Cells were allowed to proliferate for 2 days before selection allowing time for the cells to express the puromycin resistance. We utilized the selection pressure of puromycin (0.5-1 μg/ml, Sigma) and applied it directly into the cell media.

### Oxygen glucose deprivation and MTT assay

As an *in vitro* model of ischemia, oxygen glucose deprivation (OGD) was performed on the control and SDF-1-upregulated cells. iPS cells were plated into 24-well plates at 200,000 cells/well. After adhesion to the bottom of the well overnight in the incubator at normoxic conditions, cells were incubated for 3-7 hrs in the ProOx-C-chamber system (Biospherix, Redfield, NY) at 0.1-0.3% oxygen at 37°C. Control cells were maintained in normoxic conditions. After the treatment, the cells were returned to normoxic culture conditions for a 12-hour reoxygenation period. Following 12 hours of reoxygenation, the cultures were assayed with the 3-(4,5-Dimethylthiazol-2-yl)-2,5-diphenyltetrazolium bromide (MTT) assay.

MTT solution (25 μl, 5mg/mL) was added to 250 μl of cell media per well and incubated for 4 hrs at 37°C. Two hundred fifty μl of solubilization solution was added to each well and incubated overnight. The solubilization solution consisted of 10% *sodium dodecyl sulfate* (SDS) in 0.01 M HCl. Wells were read on a plate reader at 560nm to measure colorimetric optical intensity.

### Immunocytochemistry

Cultures were fixed with 4% paraformaldehyde. Cells were washed 3 times with phosphate-buffered saline (PBS) after each step. The cells were treated with -20°C ethanol/acetic acid solution, permeabilized with a 0.2% Triton-X 100 solution, and blocked with 1% cold fish gelatin (Sigma, St. Louis, MO). The cells were incubated overnight at 4°C with primary antibodies for Oct3/4 (sc-5279, 1:200; Santa Cruz Biotechnology, Santa Cruz, CA), NeuN (MAB377 1:200; Millipore), β3 Tubulin (Tuj-1, MMS-435P, 1:200; Covance, Princeton, NJ), neurofilament (AB9568, 1:100; Millipore), synaptosomal-associated protein 25 (*SNAP-25, AB5871P, 1:100;* Millipore), synapsin 1 (51-5200, 1:200; Life Technologies, Grand Island, NY), FOXG1 (1:200, Abcam, Cambridge, MA), and GFP (1:100, Polysciences, Inc., Warrington, PA). The cells were washed with PBS after the overnight antibody incubation. The corresponding secondary antibodies were applied at 1:100 for 1 hr at room temperature (Jackson ImmunoResearch, West Grove, PA), Cells were washed with PBS and Hoechst 33342 was applied (1:20,000) and washed off. The cells were cover-slipped with Vectashield mounting media (Vector Laboratories, Burlingame, CA).

### Electrophysiological recordings

iPS cell-derived neurons were recorded with whole-cell patch clamp 10 days after harvest using an EPC9 amplifier (HEKA, Elektronik, Lambrecht, Germany) at room temperature. The recording external solution consisted of (mM) 135 NaCl, 5 KCl, 1 MgCl_2_, 2 CaCl_2_, 10 HEPES, and 10 Glucose at a pH of 7.4. The electrodes used for recording were pulled from borosilicate glass pipettes (Sutter Instrument, USA) had a tip resistance between 5 and 8 MΩ when filled with the internal solution (mM): 140 KCl, 2 MgCl_2_, 1 CaCl_2_, 2 Na_2_ATP, 10 EGTA, and 10 HEPES at a pH of 7.2. The series resistance was compensated by 60-80%. Linear leak and residual capacitance currents were subtracted on-line using a P/6 protocol. Action potentials (APs) were recorded under current-clamp mode using Pulse software (HEKA, Elektronik). Tetrodotoxin (TTX, 1 μM) was applied to block voltage-gated inward sodium currents. Data were filtered at 3 KHz and digitized at sampling rates of 20 KHz. AP amplitude was determined by measurement from the initial threshold to the peak of AP upstroke.

### Reverse transcriptase-polymerase chain reaction (RT-PCR)

The total mRNA was isolated from the pluripotent, progenitor, and differentiated cells with Trizol (Invitrogen Life Technologies, 250 μl per dish). 50 μl of Choloroform was added to each sample. The samples were centrifuged and the upper aqueous phase was collected and RNA was precipitated with isopropyl alcohol. The samples were centrifuged and washed two times with 75% ethanol. The RNA pellet was dried and resuspended in DEPC-treated water. cDNA was created from the RNA samples using the High Capacity RNA-to-cDNA kit™ (Applied Biosystems Life technologies, Grand Island, NY). PCR reactions consisted of a mixture of Taq buffer (New England Biolabs, Ipswich, MA), forward and reverser primers (listed below) dNTP (10mM), Taq polymerase, water and cDNA. Electrophoresis was used to run DNA samples on a 1.8% agarose gel. Band intensity was quantified using ImageJ software (NIH, Bethesda, MD).

Primer pairs:

18S: GACTCAACACGGGAAACCTC (forward), ATGCCAGAGTCTCGTTCGTT (reverse)

Bcl-2: GCTGTGAGGGAGCAAGAATC (forward), GGTCAAGAGGGAGTGTTGGA (reverse)

SDF-1α: GCTCTGCATCAGTGACGGTA (forward), CCAGGTACTCTTGGATCCAC (reverse)

### Focal ischemia surgery, iPS-NPC transplantation, and tissue collection

Adult C57BL/6 male mice (25-28 g, 2-3 month-old) were housed in a climate-controlled room with a 12-hr light-dark cycle with free access to water and food. All animal procedures were approved by the Institutional Animal Care and Use Committee (IACUC) at Emory University. A total number of 57 mice were tested in this study. The mortality rate due to surgery and anesthesia failure were generally less than 10% in our animal experiments.

To induce focal cerebral ischemia in the adult mice, the mice were anesthetized with 3% isoflurane and maintained using 1.5% isoflurane supplemented with regular air during surgery. Surgery commenced when the mouse showed no response to pinches to test reflexes. After a skin incision, the skull was drilled over the distal branches of the right middle cerebral artery. The distal branches of the right middle cerebral artery (MCA) were permanently occluded and bilateral common carotid arteries (CCAs) were ligated for 7 min to produce an ischemia in the right sensorimotor cortex. Sham animals received skin incisions, but no arterial occlusions. Mice received solution control and cell transplantation 7 days after stroke (n=8 mice per group for immunostaining examinations, n=5 per group in behavioral tests). The mice were anesthetized as above until they were in a deep anesthetic state verified through testing the tail and paw pinch reflex. The incision over the right side of stroke was opened and the skull was thinned over the area to be injected approximately 3mm lateral to bregma. iPS-NPCs (100,000 or 300,000 cells as low and high dose groups) were transplanted in 4 μl, divided into 2 injection sites in the peri-infarct region at a depth of 900 μm from the dura to the tip of the needle. Injections were delivered slowly with a Hamilton’s syringe. Ischemia injured tissue can be identified by fainter image than the surrounding tissue. Cells were injected less than 0.5 mm from the border of the core region. The presence and location of the cells was later confirmed with immunostaining. Stroke only control animals received 2 x 2 μl injections of SATO media. On the day of transplantation and thereafter, mice received a daily intraperitoneal (i.p.) injection of Bromodeoxyuridine (BrdU; 50 mg/kg; Sigma). At 14 days after transplantation, mice were euthanized by overdose isoflurane before decapitation to dissect tissue to flash freeze. Some animals were anesthetized for perfusion fixation for immunohistochemistry. Once the mouse was unresponsive to the pinch reflex, an incision was made along the diaphragm and two incisions were made through the ribcage on both sides of the mouse. The sternum and ribs were pulled back to expose the heart. A needle with tubing connected to a perfusion pump was inserted into the right atrium and a small cut was made in the left ventricle. Saline was pumped through the vasculature to clear the blood and then it was replaced with 10% buffered formalin. Perfusion was complete once the animal was stiff. The brain was then dissected out and post-fixed with sucrose and formalin.

### Immunohistochemistry

Fresh frozen or formalin-perfused brains were sectioned at 10 μm on a cryostat microtome at -20°C. To avoid double-counting a cell in two adjacent sections, design-based stereology was used. Every tenth section was collected such that adjacent tissues were more than 100 μm apart. Tissues were then fixed with 10% buffered formalin, washed, and treated a -20°C ethanol/acetic acid solution. Tissues were permeabilized with a 0.2% Triton-X 100 solution, and blocked with 1% fish gel (Sigma). Tissues were washed three times with PBS after each step. After blocking, tissues were incubated overnight at 4°C with primary antibodies for BrdU (1:400; AbD Serotec, Hercules, CA), NeuN (1:200; Millipore), Glut-1 (1:200, Millipore), and GFP (1:100, Polysciences, Inc., Warrington, PA). After washing with PBS, secondary antibodies conjugated to fluorophores were used to visualize BrdU (donkey anti-rat Cy3, 1:300; Jackson ImmunoResearch), NeuN (donkey anti-mouse Cy5, 1:200), and Collagen IV (donkey anti-goat AlexaFluor 488, 1:200; Invitrogen Life Technologies). The secondary antibodies (Jackson ImmunoResearch) were applied and incubated on the tissue for 1 hr at room temperature and subsequently washed 3 times with PBS and coverslipped with Vectashield. Using fluorescent microscopy, 10x pictures were captured from 4 areas 700-900μm from the edge of the injury. Six tissue sections from each animal brain were quantified. NeuN/BrdU, Glut-1/BrdU, and GFP/BrdU co-labeled cells were quantified. For each animal group (sham, stroke, stroke + control cells, stroke + SDF-1 cells), n = 8 mice. Not all animals were subjected to the same staining assays.

### Top scan behavioral tracking

The Top Scan software from CleverSys, Inc (Reston, VA) was used to track animal walking distance, and velocity in a testing chamber (50cm x 50cm). Seven and 14 days after transplantation, an individual mouse was placed into the testing chamber and recorded by the tracking system for 1 hour. For each animal group n=5 mice. The data was analyzed and graphed using the Prism Graphpad software (GraphPad Software, Inc., La Jolla, CA).

### Statistical analysis

Student’s t-test was used for single comparisons. One-way and two-way analysis of variance (ANOVA) followed by Bonferroni’s post-hoc analysis was used for multiple comparisons. Significance was defined as a p-value that was less than 0.05 and standard error of the mean (SEM) was reported with the means. Data are presented as Mean±SEM.

## Abbreviations

iPS cell: induced pluripotent stem cell
iPS-NSC: iPS cell-derived neural progenitor cells
SDF-1α: stromal cell-derived factor-1α
SDF-1ɑ-iPS-NPCs: SDF-1ɑ-overexpressing iPS cell-derived neural progenitor cells
RA: retinoic acid
